# Risk of Diabetes Mellitus Among Medicaid Beneficiaries in Hawaii

**DOI:** 10.5888/pcd14.170095

**Published:** 2017-11-22

**Authors:** Dongmei Li, Chuan C. Chinn, Ritabelle Fernandes, Christina M.B. Wang, Myra D. Smith, Rebecca Rude Ozaki

**Affiliations:** 1 University of Rochester School of Medicine and Dentistry, Rochester, New York; 2 University of Hawai’i at Mānoa, Honolulu, Hawai’i

## Abstract

**Introduction:**

Medicaid is the largest primary health insurance for low-income populations in the United States, and it provides comprehensive benefits to cover treatment and services costs for chronic diseases, including diabetes. The standardized per capita spending on diabetes by Medicare beneficiaries enrolled in the fee-for-service program in Hawaii increased from 2012 to 2015. We examined the difference in odds of diabetes between Medicaid and non-Medicaid populations in major racial/ethnic groups in Hawaii.

**Methods:**

We used data from 2013 through 2015 from the Hawaii Behavioral Risk Factor Surveillance System in this cross-sectional study to compare the difference in risk for self-reported diabetes between Medicaid (n = 1,889) and non-Medicaid (n = 17,207) beneficiaries. We used multivariate logistic regression models that could accommodate the complex sampling design to examine the difference in odds of diabetes between the 2 populations.

**Results:**

In Hawaii, the Medicaid population was younger, was less educated, had more health impairments, and was more likely to be obese and Native Hawaiian/Other Pacific Islander (NH/OPI) than the non-Medicaid population. The unadjusted prevalence of diabetes in the Medicaid population in Hawaii was higher than that for the non-Medicaid population (10.3% vs 8.9%, *P* = .02). After adjusting for confounding variables, the odds of diabetes in the Medicaid population was still significantly higher than those in the non-Medicaid population (adjusted odds ratio [AOR] = 1.75; 95% confidence interval [CI], 1.33–2.31). Adjusted analysis stratified by race/ethnicity showed that non-Hispanic Asian (AOR = 2.23; 95% CI, 1.31–3.78) and NH/OPI (AOR = 3.17; 95% CI, 1.05–9.54) Medicaid beneficiaries had significantly higher odds of diabetes than their non-Medicaid counterparts.

**Conclusion:**

The odds of diabetes was significantly higher among the Hawaii Medicaid population than among the non-Medicaid population. Diabetes prevention programs should address the challenges and barriers that the Medicaid population faces. Our findings can be used to promote culturally competent diabetes education programs.

## Introduction

Diabetes is a complex disease that affects nearly 400 million people worldwide and in 2014 was the seventh leading cause of death in the United States (1,2). The prevalence of diabetes has steadily increased since 1990. In 2011–2012, the estimated prevalence was 12% to 14% among US adults, with a higher prevalence among blacks, Asians, Native Hawaiian/Other Pacific Islanders (NH/OPIs), and Hispanics (3,4). In 2016, more than 29 million Americans were living with diabetes, and 86 million had prediabetes (5). In 2012, the total estimated cost of diagnosed diabetes was $245 billion (6).

Low-income populations have a disproportionately high prevalence of diabetes (7). Medicaid, as the nation’s largest primary health insurance for low-income populations, plays an important role in financial support by providing comprehensive benefits to cover treatment and services costs for chronic diseases (8). The standardized per capita spending on diabetes by Medicare beneficiaries enrolled in the fee-for-service program in Hawaii, of which 23% were dual eligible, increased from $9,960 in 2012 to $10,425 in 2015 (9). Beginning in 2014, the Patient Protection and Affordable Care Act (ACA, PL. 111–148 as amended) allowed states to expand Medicaid eligibility to cover all individuals living up to 133% of the federal poverty rate, which further increased the size of the nation’s Medicaid population and associated financial costs (10).

The annual Behavioral Risk Factor Surveillance System (BRFSS) conducted in Hawaii traditionally does not distinguish between Medicaid and non-Medicaid populations (11). In the 2013–2015 BRFSS administered by the Hawaii State Department of Health, an additional question was included about participants’ Medicaid status. The purpose was to describe differences in sociodemographic characteristics between Medicaid and non-Medicaid populations and evaluate the risk of diabetes for the Medicaid population to provide fundamental knowledge for future diabetes prevention and management programs in Hawaii. Medicaid is a jointly funded, federal–state health insurance program (12). Federal law requires states to cover certain groups with Medicaid, including low-income families, older adults, qualified pregnant women and their children, and individuals receiving Supplemental Security Income. Although results of epidemiological studies show a significant association between low socioeconomic status (SES) and diabetes, the Medicaid status question added to the Hawaii BRFSS survey provides more information on the association between diabetes risk and those with low SES who are insured (13,14).

A review of the literature indicated that little research had investigated the association between Medicaid status and risk of diabetes in the Hawaii adult population. We examined the difference in odds of diabetes between Medicaid and non-Medicaid populations in major racial/ethnic groups in Hawaii.

## Methods

### Data source

BRFSS survey data from 2013 through 2015 were obtained from the Hawaii Department of Health through a data use agreement. The BRFSS is a cross-sectional telephone survey of noninstitutionalized civilians in the United States aged 18 years or older (15). The survey is federally funded by the Centers for Disease Control and Prevention and is conducted annually by the Hawaii Department of Health (16). Funding for the additional question “Do you have Medicaid or Med-QUEST?” was supported by the Hawaii Patient Reward and Incentives to Support Empowerment (HI-PRAISE) project through the Centers for Medicare & Medicaid Services, Medicaid Incentives for Prevention of Chronic Diseases program. This study was approved by the University of Hawaii institutional review board. Med-QUEST is a division in the State Department of Human Services that provides eligible low-income adults and children access to health and medical coverage through managed care plans. The term may be known and used interchangeably by the state’s Medicaid beneficiaries.

A multistage cluster design was used in the BRFSS to produce a nationally representative sample. Each respondent had a corresponding final weight based on the respondent’s probability of being selected into the BRFSS sample and a poststratification factor to ensure consistency of the age and racial/ethnic distribution between the BRFSS and US census data. For combined BRFSS 2013–2015 data, the final weight was calculated as the average of the final weight in each year’s data. Requested BRFSS data included core sections and state modules on prediabetes, diabetes, and cardiovascular health. The core sections included in the data analysis were health status, number of healthy days and health-related quality of life, health care access, sleep status, hypertension awareness, cholesterol awareness, chronic health conditions, alcohol consumption, fruit and vegetable consumption, exercise, immunization status, and demographic information. A participant with diabetes was defined by a yes response to the question “(Ever told) you have diabetes?” Survey respondents with gestational diabetes were excluded from the study population.

### Data analysis

Weighted frequency distributions and cross-tabulations were used to describe the demographic and health characteristics of the BRFSS 2013–2015 samples, stratified by Medicaid status. Wald χ^2^ tests were used to assess the bivariate association between demographic characteristics and Medicaid status. Bivariate and multivariate logistic regression analyses were used to examine the association between Medicaid status and risk for diabetes, controlling for the independent effects of sex, body mass index (BMI), age, race/ethnicity, marital status, checkup within past year, exercise, immunization status, and alcohol consumption. Stratified analyses using multivariate logistic regression models were conducted for Medicaid and non-Medicaid groups separately to examine the group differences in diabetes risks. Stratified analyses were also conducted for racial/ethnic groups (non-Hispanic white, Asian, and NH/OPIs). The effects of other independent variables on risk of diabetes were compared between the Medicaid and non-Medicaid groups and among the 3 non-Hispanic racial groups. The Hosmer and Lemeshow purposeful model selection method was used to select the confounding variables used in the multivariate logistic regression models (17). All analyses were conducted using SAS version 9.4 (SAS Institute Inc) statistical analysis software, using procedures for survey data analysis with strata, primary sampling units, and final sampling weights to account for the complex sampling design. Adjusted odds ratios (AORs) and their 95% confidence intervals (CIs) were used to quantify the strength of the association between risk factors and diabetes.

## Results

In the combined Hawaii 2013–2015 BRFSS samples, 19,096 people responded to the Medicaid question, among which 1,889 (9.9%) self-identified as a Medicaid beneficiary. After adjusting the complex sampling design weight, the proportion of Medicaid population was 8.8% during the 3-year period. Most demographic and health characteristics were significantly different between the Medicaid and non-Medicaid populations (Table 1). The Medicaid population was more likely to be female, to be obese, to be younger than 45 years, to be NH/OPI, and to identify as being 2 or more races or of Hispanic ethnicity, and was less likely to be married. In addition, the Medicaid population was less likely than the non-Medicaid population to have more than a high school education, own a home, ever serve on active duty, be employed, and have an annual household income of more than $35,000. They indicated more activity limitations, blindness, and health problems requiring special equipment and were less likely to participate in any physical activities or exercise. Compared with the non-Medicaid population, the Medicaid population also indicated that they had more difficulty concentrating, remembering, or making decisions resulting from a physical, mental, or emotional condition; more difficulty in doing errands alone, such as visiting a doctor’s office or shopping; more difficulty in dressing or bathing; and more serious difficulty in walking or climbing stairs. Respondents with Medicaid also were more likely to have chronic illnesses such as depression; asthma; chronic obstructive pulmonary disease, emphysema, or chronic bronchitis; and myocardial infarction, and they reported more days with poor physical and mental health (6.8 vs 4.0 days during the past 30 days). Fewer Medicaid beneficiaries had had an adult influenza shot or spray during the past 12 months or been tested for high blood glucose or diabetes in the past 3 years. Furthermore, compared with the non-Medicaid population, the Medicaid population had fewer number of days in past 30 days to have had an alcoholic beverage (3.5 vs 5.1), but they had a higher daily average alcohol consumption (4.2 drinks per day vs 2.8 drinks per day in past 30 days) and had a higher level of binge drinking (2.9 times vs 1.7 times during the past 30 days having ≥5 drinks for men or ≥4 drinks for women on an occasion).

**Table 1:** Sample Characteristics, by Medicaid Status, Hawaii Behavioral Risk Factor Surveillance System, 2013–2015^a^

Characteristic	Medicaid		Non-Medicaid		*P* Value
	No. (n = 1,889)	Value (n = 84,635)	No. (n = 17,207)	Value (n = 87,2301)	
**Sex**					
Male	786	35,761 (42.3)	8,145	438,923 (50.3)	<.001
Female	1,103	48,874 (57.7)	9,062	433,378 (49.7)	
**Body mass index**					
Normal	746	32,830 (39.3)	7,297	361,449 (43.2)	<.001
Overweight	581	26,168 (31.3)	5,796	291,709 (34.9)	
Obese	535	24,497 (29.3)	3,478	182,729 (21.9)	
**Age, y**					
18–44 (young)	840	53,860 (63.9)	5,056	376,288 (43.7)	<.001
45–64 (middle-aged)	676	20,717 (24.6)	6,467	294,807 (34.2)	
≥65 (senior)	364	9,706 (11.5)	5,478	190,047 (22.1)	
**Race/ethnicity**					
White, non-Hispanic	498	17,708 (21.1)	5,522	229,108 (26.6)	<.001
Black, non-Hispanic	9	688 (0.8)	121	11,977 (1.4)	
American Indian/Alaska Native, non-Hispanic	4	215 (0.3)	42	2,385 (0.3)	
Asian, non-Hispanic	315	17,214 (20.6)	5,460	350,034 (40.6)	
NH/OPI, non-Hispanic	118	5,939 (7.1)	479	22,113 (2.6)	
Other race, non-Hispanic	16	329 (0.4)	78	2,981 (0.3)	
Two or more races, non-Hispanic	687	28,710 (34.3)	4,067	167,681 (19.4)	
Hispanic	219	12,930 (15.4)	1,238	76,026 (8.8)	
**Marital status**					
Married	477	22,249 (26.3)	8,968	481,230 (55.5)	<.001
Divorced	391	11,362 (13.4)	2,209	83,625 (9.6)	
Widowed	132	3,735 (4.4)	1,677	59,122 (6.8)	
Separated	59	2,898 (3.4)	248	12,601 (1.5)	
Never married	748	40,553 (48.0)	3,571	207,407 (23.9)	
A member of an unmarried couple	76	3,706 (4.4)	428	23,183 (2.7)	
**Education**					
High school or less	163	16,038 (19.0)	653	72,878 (8.4)	<.001
More than high school	1,725	68,574 (81.0)	16478	794,292 (91.6)	
**Home ownership**					
Own	501	25,322 (30.1)	10,474	566,345 (66.4)	<.001
Rent	1,001	40,497 (48.2)	4,767	200,784 (23.6)	
Other arrangements	378	18,261 (21.7)	1,630	85,326 (10.0)	
**Ever served on active duty**	166	5,761 (6.8)	2,742	138,190 (15.9)	<.001
**Employment status**					
Employed for wages	499	25,138 (29.8)	8,065	467,729 (54.0)	<.001
Self-employed	278	11,334 (13.4)	1,960	88,781 (10.2)	
Out of work for 1 year or more	156	8,325 (9.9)	319	15,830 (1.8)	
Out of work for less than 1 year	104	6,324 (7.5)	334	19,132 (2.2)	
A homemaker	122	6,648 (7.9)	589	35,240 (4.1)	
A student	104	6,999 (8.3)	455	37,138 (4.3)	
Retired	320	8,004 (9.5)	4,878	179,144 (20.7)	
Unable to work	301	11,587 (13.7)	513	23,785 (2.7)	
**Annual household income, $**					
<10,000	454	18,127 (23.6)	575	29,377 (3.8)	<.001
10,000–14,999	253	9,134 (11.9)	573	23,615 (3.0)	
15,000–19,999	282	12,575 (16.3)	926	44,437 (5.7)	
20,000–24,999	232	11,753 (15.3)	1,146	56,240 (7.2)	
25,000–34,999	209	11,078 (14.4)	1,718	85,284 (11.0)	
35,000–49,999	131	5,836 (7.6)	2,341	110,957 (14.3)	
50,000–74,999	75	3,247 (4.2)	2,791	135,658 (17.5)	
≥75,000	98	5,199 (6.8)	5,419	290,703 (37.4)	
**Health problems/impairments**					
Activity limitation due to health problems	620	22,928 (27.2)	3,060	123,087 (14.6)	<.001
Blind	163	5,462 (6.5)	594	28,121 (3.3)	<.001
Health problems requiring special equipment	228	7,327 (8.7)	1,182	43,487 (5.2)	<.001
Serious difficulty concentrating, remembering, or making decisions resulting from a physical, mental, or emotional condition	347	15,462 (18.3)	1,090	50,575 (6.0)	<.001
Difficulty doing errands alone such as visiting a doctor’s office or shopping	262	10,509 (12.4)	752	32,215 (3.9)	<.001
Difficulty dressing or bathing	132	4,703 (5.6)	360	13,917 (1.7)	<.001
Serious difficulty walking or climbing stairs	387	13,809 (16.3)	1,787	72,121 (8.6)	<.001
**Exercise (physical activity)**					
Participate in any physical activities or exercises	1,406	63,112 (74.6)	13,200	657,337 (79.2)	.002
**Chronic health conditions**					
Depression	464	19,613 (23.2)	1,993	87,489 (10.1)	<.001
Asthma	416	22,630 (26.9)	2,561	135,365 (15.6)	<.001
Chronic asthma	296	15,183 (68.6)	1,502	76,799 (58.1)	.01
COPD, emphysema, or chronic bronchitis	146	5,279 (6.3)	831	35,602 (4.1)	.003
Skin cancer	111	2,623 (3.1)	1,348	41,500 (4.8)	<.001
Other types of cancer	138	4,530 (5.4)	1,290	50,355 (5.8)	.57
Heart attack or myocardial infarction	106	3,743 (4.5)	655	26,148 (3.0)	.02
Kidney disease	92	3,820 (4.5)	719	30,103 (3.5)	.18
Angina or coronary heart disease	74	2,380 (2.8)	626	26,017 (3.0)	.73
Stroke	99	3,012 (3.6)	569	24,982 (2.9)	.16
Arthritis	482	16,135 (19.2)	4,289	175,258 (20.2)	.44
**Health care access**					
Checkup within past year	1,251	54,048 (64.1)	11,925	594,916 (68.4)	.08
**Immunization**					
Adult influenza shot/spray in past 12 months	713	30,614 (36.5)	7,354	362,879 (45.5)	<.001
Pneumonia shot ever	539	22,151 (31.4)	5,231	223,993 (32.5)	.53
**Diabetes screening**					
Tested for high blood glucose or diabetes in past 3 years	584	22,145 (46.5)	5,139	242,052 (52.2)	.01
**Alcohol consumption in past 30 days, weighted mean (SD)**					
No. of days had an alcoholic beverage	1,882	3.5 (0.2)	16,505	5.1 (0.1)	<.001
Average no. of alcoholic drinks per day	772	4.2 (0.3)	8,624	2.8 (0.1)	<.001
Largest no. of alcoholic drinks on a single occasion	760	6.1 (0.4)	8,500	4.2 (0.1)	<.001
Binge drinking^b^	770	2.9 (0.4)	8,605	1.7 (0.1)	.004
**No. of days with poor physical and mental health, weighted mean (SD)**	1,100	6.8 (0.4)	7,576	4.0 (0.1)	<.001

Abbreviations: COPD, chronic obstructive pulmonary disease; NH/OPI, Native Hawaiian/Other Pacific Islander; SD, standard deviation.

a Values presented as weighted no. (%), unless otherwise indicated.

b Binge drinking was number of times during the past 30 days that men had consumed 5 or more drinks and women had consumed 4 or more drinks on 1 occasion.

The prevalence of diabetes in the Medicaid population was 10.3%, compared with 8.9% in the non-Medicaid population, and the unadjusted difference of diabetes prevalence was significant (*P* = .02). The adjusted odds of diabetes among the Medicaid population was significantly higher than the odds among the non-Medicaid population (AOR = 1.75; 95% CI, 1.33–2.31) (Table 2). The adjusted odds of diabetes was significantly higher among all other races/ethnicities, particularly among Asians and NH/OPIs, than among non-Hispanic whites.

**Table 2:** Adjusted Odds of Diabetes, by Medicaid Status, Hawaii Behavioral Risk Factor Surveillance System, 2013–2015^a^

Variables	All	Medicaid	Non-Medicaid
	Adjusted Odds Ratio (95% Confidence Interval)		
**Medicaid beneficiary**	1.75 (1.33–2.31)	—	—
**Sex**			
Female	1 [Reference]		
Male	1.21 (1.02–1.44)	1.44 (0.85–2.45)	1.18 (0.98–1.42)
**Body mass index**			
Normal	1 [Reference]		
Overweight	2.02 (1.61–2.53)	2.86 (1.54–5.32)	1.94 (1.52–2.47)
Obese	4.34 (3.44–5.47)	3.04 (1.70–5.44)	4.59 (3.58–5.89)
**Age, y**			
18–44 (young)	1 [Reference]		
45–64 (middle-aged)	3.47 (2.62–4.60)	3.29 (1.84–5.90)	3.60 (2.61–4.97)
≥65 (senior)	4.05 (2.95–5.56)	2.48 (1.18–5.22)	4.26 (2.99–6.07)
**Race/ethnicity**			
White, non-Hispanic	1 [Reference]		
Asian, non-Hispanic	2.51 (2.04–3.09)	3.90 (1.99–7.65)	2.43 (1.95–3.03)
NH/OPI, non-Hispanic	2.80 (1.86–4.20)	4.68 (1.91–11.49)	2.41 (1.56–3.71)
Two or more races, non-Hispanic	2.31 (1.84–2.89)	2.60 (1.40–4.83)	2.28 (1.80–2.90)
Hispanic	2.22 (1.61–3.07)	1.97 (0.92–4.21)	2.31 (1.63–3.29)
**Marital status**			
Married	1 [Reference]		
Divorced	0.75 (0.59–0.95)	1.05 (0.57–1.93)	0.73 (0.56–0.95)
Widowed	1.09 (0.85–1.39)	0.94 (0.37–2.42)	1.10 (0.86–1.42)
Separated	0.66 (0.33–1.33)	0.87 (0.25–3.05)	0.63 (0.26–1.49)
Never married	0.73 (0.56–0.94)	0.88 (0.46–1.69)	0.68 (0.51–0.90)
Member of an unmarried couple	0.60 (0.29–1.22)	0.95 (0.22–4.16)	0.52 (0.24–1.12)
**Checkup within past year**	1.92 (1.51–2.46)	1.76 (0.93–3.33)	1.95 (1.51–2.53)
**Participate in any physical activities or exercises**	0.63 (0.52–0.77)	0.64 (0.40–1.03)	0.63 (0.51–0.78)
**Immunization**			
Adult influenza shot/spray during past 12 months	1.34 (1.13–1.60)	1.08 (0.67–1.74)	1.38 (1.14–1.67)
Pneumonia shot ever	2.51 (2.06–3.05)	2.60 (1.50–4.49)	2.52 (2.04–3.11)
**Days in past 30 days had an alcoholic beverage**	0.96 (0.95–0.98)	0.98 (0.94–1.01)	0.96 (0.95–0.98)

Abbreviations: —, not applicable; NH/OPI, Native Hawaiian/Other Pacific Islander.

a All variables in the multivariate logistic regression models were included in the table. For all participants, the sample size used in the analysis was 13,874 with a weighted sample size of 664,939. For the Medicaid subgroup, the sample size used in the analysis was 1,416 with a weighted sample size of 61,587. For the non-Medicaid subgroup, the sample size used in the analysis was 12,483 with a weighted sample size of 604,505.

### Subgroup analysis by Medicaid status

Although men had slightly higher odds of diabetes than did women in the overall sample, the difference between sexes was not significant in either the Medicaid group or non-Medicaid group (Table 2). High BMI was a significant risk factor in both groups. The odds of diabetes for middle-aged adults (aged 45 to 64) was significantly higher than that for young adults (aged 18 to 44 years) in both groups. Asians, NH/OPIs, and participants identifying as 2 or more non-Hispanic races had higher odds of diabetes than non-Hispanic whites in both the Medicaid and non-Medicaid groups. However, Hispanics had significantly higher odds only of diabetes in the non-Medicaid group (AOR = 2.31; 95% CI, 1.63–3.29) but not in the Medicaid group (AOR = 1.97; 95% CI, 0.92–4.21). The positive effect of exercise was observed in the non-Medicaid population only. Alcohol consumption had a significant protective effect on odds of diabetes in the non-Medicaid population only (AOR = 0.96; 95% CI, 0.95–0.98).

### Subgroup analysis by race

The adjusted odds of diabetes was not significantly different between the Medicaid population and non-Medicaid population among non-Hispanic whites (AOR = 1.32; 95% CI, 0.79–2.22) (Table 3). However, the adjusted odds of diabetes was significantly higher in the Medicaid population than non-Medicaid population among Asians (AOR = 2.23; 95% CI, 1.31–3.78) and NH/OPIs (AOR = 3.17; 95% CI, 1.05–9.54). Sex was not significant for any of the 3 racial/ethnic groups. Being overweight or obese was a risk factor among all 3 racial/ethnic groups; AORs ranged from 1.80 to 7.45. The adjusted odds among white seniors (aged ≥65 y) (AOR = 6.26; 95% CI, 3.01–13.02) and middle-aged whites (AOR = 4.22; 95% CI, 1.99–8.92) was higher than that among young whites. Both middle-aged Asians and Asian seniors had higher odds of diabetes than did young Asians, but the AORs were similar among middle-aged Asians (AOR = 3.21; 95% CI, 1.86–5.53) and Asian seniors (AOR = 2.97; 95% CI, 1.65–5.36). Middle-aged NH/OPIs (AOR = 2.54; 95% CI, 1.06–6.06) had significantly higher odds of diabetes than did young NH/OPIs, although no significant difference was found between NH/OPI seniors and young NH/OPIs (AOR = 3.13; 95% CI, 0.95–10.30).

**Table 3:** Adjusted Odds of Diabetes, by Race/Ethnicity, Hawaii Behavioral Risk Factor Surveillance System, 2013–2015^a^

Variables	Non-Hispanic White	Non-Hispanic Asian	Native Hawaiian/Other Pacific Islander, Non-Hispanic
	Adjusted Odds Ratio (95% Confidence Interval)		
**Medicaid status**			
Non-beneficiary	1 [Reference]		
Beneficiary	1.32 (0.79–2.22)	2.23 (1.31–3.78)	3.17 (1.05–9.54)
**Sex**			
Female	1 [Reference]		
Male	1.35 (0.95–1.91)	1.21 (0.91–1.62)	1.93 (0.79–4.67)
**Body mass index**			
Normal	1 [Reference]		
Overweight	1.80 (1.17–2.77)	1.94 (1.41–2.67)	7.17 (1.85–27.75)
Obese	5.23 (3.37–8.12)	4.11 (2.85–5.93)	7.45 (2.06–26.96)
**Age, y**			
18–44 (young)	1 [Reference]		
45–64 (middle-aged)	4.22 (1.99–8.92)	3.21 (1.86–5.53)	2.54 (1.06–6.06)
≥65 (senior)	6.26 (3.01–13.02)	2.97 (1.65–5.36)	3.13 (0.95–10.30)
**Marital status**			
Married	1 [Reference]		
Divorced	1.01 (0.64–1.62)	0.62 (0.41–0.94)	1.46 (0.49–4.31)
Widowed	0.76 (0.44–1.32)	1.46 (1.03–2.07)	2.13 (0.55–8.25)
Separated	0.66 (0.23–1.93)	1.01 (0.24–4.32)	0.20 (0.02–2.63)
Never married	0.84 (0.48–1.49)	0.69 (0.46–1.04)	0.73 (0.25–2.14)
Member of an unmarried couple	0.86 (0.32–2.31)	<.001	1.66 (0.16–17.63)
**Checkup status**			
No checkup within past year	1 [Reference]		
Checkup within past year	2.45 (1.47–4.08)	1.98 (1.31–2.98)	2.53 (0.96–6.66)
**Physical activity**			
Do not participate in any physical activities or exercises	1 [Reference]		
Participate in any physical activities or exercises	0.63 (0.43–0.91)	0.62 (0.45–0.85)	0.88 (0.39–1.98)
**Immunization**			
Adult influenza shot/spray during past 12 months	1.37 (0.96–1.96)	1.20 (0.90–1.61)	1.07 (0.51–2.22)
Pneumonia shot ever	3.42 (2.33–5.03)	2.50 (1.79–3.48)	3.73 (1.75–7.92)
**Alcohol consumption**			
Did not have an alcoholic beverage in past 30 days	1 [Reference]		
Had an alcoholic beverage in past 30 days	0.96 (0.95–0.98)	0.97 (0.95–0.99)	0.98 (0.93–1.03)

a All variables in the multivariate logistic regression models were included in the table. For the white subgroup, the sample size used in the analysis was 4,783 with a weighted sample size of 186,600. For the Asian subgroup, the sample size used in the analysis was 4,151 with a weighted sample size of 256,054. For the Native Hawaiian/Other Pacific Islander subgroup, the sample size used in the analysis was 413 with a weighted sample size of 19,042.

A checkup within the past year was associated with elevated odds of diabetes among both whites (AOR = 2.45; 95% CI, 1.47–4.08) and Asians (AOR = 1.98; 95% CI, 1.31–2.98); however, this association was not significant among NH/OPIs (AOR = 2.53; 95% CI, 0.96–6.66). Physical activity was a protective factor for diabetes among whites (AOR = 0.63; 95% CI, 0.43–0.91) and Asians (AOR = 0.62; 95% CI, 0.45–0.85) but was not significant among NH/OPIs (AOR = 0.88; 95% CI, 0.39–1.98). More alcohol consumption in the past 30 days was significantly associated with reduced odds of diabetes among both whites (AOR = 0.96; 95% CI, 0.95–0.98) and Asians (AOR = 0.97; 95% CI, 0.95–0.99) but was not significant among NH/OPIs (AOR = 0.98; 95% CI, 0.93–1.03).

## Discussion

Few studies have examined the difference in health behavior risk factors between the Medicaid and non-Medicaid populations in Hawaii, and we found no research on adults with Medicaid in relation to diabetes. A study on children and adolescents with type 1 or type 2 diabetes that included participants from Hawaii found that worse health-related quality of life was associated with a primary insurance source of Medicaid or other government-funded insurance (18).

Our study was the first to compare the adult Medicaid and adult non-Medicaid populations in Hawaii on demographic characteristics and association with diabetes after the Medicaid question was added. Although studies on the association between low SES and diabetes have been conducted using various SES indicators such as income, occupation, or educational level, Medicaid status is a more comprehensive indicator for those with low SES and insured in the Hawaii population (19,20). The Medicaid status question provided an opportunity to examine the associations between Medicaid status, race/ethnicity, and diabetes in the Hawaii population. Our findings may be of interest to other states’ Medicaid population. We showed significant differences between Medicaid and non-Medicaid populations on demographic characteristics, chronic health conditions, functional difficulties, physical activities, and drinking behaviors. Notable was that 64% of Medicaid beneficiaries in Hawaii were young adults (aged 18–44 y), compared with 44% in the non-Medicaid group. Additionally, unlike the non-Medicaid members whose odds of diabetes increased with age, the middle-aged Medicaid beneficiaries demonstrated the highest odds, which may have resulted from the small sample size of senior Medicaid beneficiaries. Finally, in Hawaii, the Medicaid population had significantly higher odds of diabetes than the non-Medicaid population after adjusting for potential confounding variables.

Stratified analysis by Medicaid status demonstrated different risk factors associated with elevated risk of diabetes. Having received an influenza vaccination in the past 12 months was significantly associated with diabetes in the non-Medicaid population; this finding is a correlation and does not imply causation or an increased odds of diabetes. It may indicate a marker of increased interaction with the medical system and thus increased opportunity for diabetes detection and diagnosis. It is current standard of practice and recommended by the American Diabetes Association (ADA) that people with diabetes receive the influenza vaccine annually.

Our study showed that racial/ethnic minority populations in Hawaii had significantly higher diabetes risk. The risk remained elevated for Asian and NH/OPI Medicaid beneficiaries compared with their non-Medicaid counterparts. This finding was consistent with data showing that NH/OPIs have significant disparities in health risks and outcomes and suggests that outreach efforts should be conducted for Asian and NH/OPI Medicaid beneficiaries (21).

A limitation of this study was that BRFSS data are self-reported; therefore, only people who self-reported that they have been told they have diabetes were included, and those who were undiagnosed were not included. Thus, detection bias could affect the associations between risk factors and diabetes. BRFSS survey data are also limited by the survey questions. For example, the BRFSS asked only whether the participant had any exercise in the past 30 days and did not ask about frequency or intensity of the exercise. Moderate physical activity is inversely associated with diabetes (22). The ADA recommends regular physical activity to promote a healthy lifestyle in self-management of diabetes. The nonsignificant association between physical activity and diabetes in the Medicaid population may have resulted from the lack of information on frequency and intensity of exercise.

Medicaid beneficiaries in Hawaii were younger and had different health associations in terms of diabetes risk compared with non-Medicaid beneficiaries. These findings have implications for the health care system and for prevention and screening measures. Preventive strategies should be culturally competent and tailored to the risk factors of the Medicaid population. For example, Hula, a cultural dance program implemented in Hawaii, improved hypertension management in NH/OPIs, and could also be used to reduce BMI (23). The PILI ‘Ohana program is another example of tailoring the national Diabetes Prevention Program to NH/OPIs to reduce their body weight (24). Participants of both programs were from federally qualified health centers (FQHCs) in Hawaii serving a large Medicaid population.

Our study design may be generalized to other states interested in assessing the association between Medicaid status and diabetes or other chronic disease risk among racial/ethnic groups. However, it is important for stakeholders such as FQHCs and public health practitioners to be cognizant of the differences between the Medicaid and non-Medicaid populations and among various racial/ethnic groups in the design and implementation of effective diabetes preventive strategies that meet the unique needs stemming from patients’ Medicaid status, racial/ethnic backgrounds, and cultural heritages. Policy makers should be aware of the link between Medicaid status and increased risk for almost all chronic diseases and the burden of cost in the Medicaid population for management of diabetes or other chronic diseases. Programs with policy support for value-based care specific to reducing the burden of chronic diseases in the Medicaid population are in great need. Finally, inclusion of the Medicaid status question in the BRFSS may benefit other states that seek to study chronic disease risk factors in the Medicaid population. 
